# Diroximel fumarate confers neuroprotection *via* reduced Th1 responses and induction of the anti-oxidative Nrf2 pathway in experimental neuroinflammation

**DOI:** 10.1371/journal.pone.0355936

**Published:** 2026-08-17

**Authors:** David Freudenstein, Anna Schneeweiss, Adriana Krenz, Ralf Gold, Ralf Linker, Stefanie Haase

**Affiliations:** 1 Universitätsklinikum Regensburg Klinik und Poliklinik für Neurologie, Regensburg, Bavaria, Germany; 2 Sankt Josef-Hospital Klinik für Neurologie, Bochum, North Rhine-Westphalia, Germany; Heinrich-Heine-Universitat Dusseldorf, GERMANY

## Abstract

Inflammation and oxidative stress contribute significantly to tissue damage in multiple sclerosis. Fumaric acid esters such as Dimethyl fumarate and Diroximel fumarate (DRF) exhibit immunomodulatory and antioxidative properties, although their mechanisms remain incompletely understood. This study investigated the immunomodulatory and antioxidative mechanisms of fumaric acid esters, with a focus on DRF and its ability to confer neuroprotection in autoimmune neuroinflammation. The MOG-experimental autoimmune encephalomyelitis (EAE) mouse model was employed and DRF was administered orally. In addition to clinical scoring of EAE severity, histological analyses were performed to assess inflammatory infiltration, demyelination, and axonal density. Immune responses were examined via immunophenotyping and cytokine measurements, and mRNA expression was analyzed to evaluate Nrf2 pathway activation. DRF treatment markedly ameliorated clinical EAE severity, reduced spinal cord infiltration of T cells—particularly Th1 cells—and promoted myelin and axonal preservation. Peripheral immune cells from DRF-treated mice produced lower levels of IFN-γ and IL-17A, while Nrf2-dependent antioxidative genes were upregulated in CNS tissue. Our results demonstrate neuroprotective, immunomodulatory, and antioxidative effects of DRF, supporting its role as beneficial modulator in neuroinflammation.

## Introduction

In the last decades several therapeutic strategies for the treatment of multiple sclerosis (MS) have emerged [1] While new and aggressive treatment options for highly active disease courses have been developed to control neuroinflammatory elements of disease pathology, there is also development in the moderate efficacy sector of disease modifying therapies [2].

Diroximel fumarate (DRF) is an oral fumarate for the treatment of relapsing-remitting MS. It showed similar efficacy and safety profiles compared to the established dimethyl fumarate (DMF) [3] and a reduced risk for gastrointestinal adverse events and treatment discontinuation in comparison to DMF in the EVOLVE-MS-1 and 2 phase III studies [4]. As DMF and DRF produce bioequivalent levels of the pharmacologically active metabolite monomethyl fumarate (MMF), DRF was approved for treatment of relapsing-remitting MS without vast preclinical data.

Pharmacodynamic properties of fumaric acid esters are not fully elucidated. However, due to advancements in neuroimmunological research, several mechanisms of action for fumaric acid esters have now been identified: DMF was shown to bind to thiol groups of E3 ubiquitin ligase Keap1, leading to disinhibition of nuclear factor erythroid 2-related factor 2 (Nrf2), a transcription factor conferring antioxidative resilience. Effects include an upregulation of several targets, including NAD(P)H dehydrogenase (quinone) 1 and heme oxygenase 1, as well as long term elevation of glutathione levels after an initial glutathione depletion by thiol group binding to DMF [5]. Neuroprotective antioxidative effects correlated with a sustained survival of neuronal cells and axons *in vitro* [6] and in neuroinflammatory disease models [7,8] after DMF treatment. Concerning immunomodulatory effects, DMF treatment resulted in altered frequencies of certain T cell subsets (i.e., reduced Th1/17 response [9]), as well as a favorable skew of antigen presenting cell phenotypes and B cells via inhibition of NFkB mediated processes [10–12] and activation of the g protein coupled receptor HCAR2 [13,14]. Furthermore, DMF is described to shift microglia towards a non-inflammatory phenotype, leading to further neuroprotection [15]. In this context, fumaric acid esters are also being investigated as a therapy for neurodegenerative diseases [16].

Available evidence suggests that DMF may exert biological effects distinct from its primary metabolite MMF. In this context, immunomodulatory effects relevant to multiple sclerosis have been observed with DMF but not MMF, pointing to a potential independent contribution of DMF [12,17,18].

Considering the incompletely understood mechanisms of action of fumaric acid esters, as well as the limited preclinical data on DRF in the context of neuroinflammation, we aimed to investigate the effects of oral DRF in the murine experimental autoimmune encephalomyelitis (EAE) model of neuroinflammation.

## Materials and methods

### Animals

C57BL/6J mice were bred and housed at the central animal laboratory (ZTL), the animal care facility of the University Hospital in Regensburg (Germany) under a 12h day/night cycle and standardized environmental conditions. Mice received normal chow (ssniff V1534-300) and tap water ad libitum. All experiments were approved by the German laws for animal protection and were approved by the local ethic committees for animal welfare (Erlangen 55.2-2532-2-27-451).

Animals in experiments were closely monitored daily by trained personnel. Supportive measures included provision of moistened food and water dishes on the cage floor to facilitate access for animals with paraparesis. Housing conditions were adapted as required to maintain welfare, including adjustments to enrichment and cage hygiene. All procedures were performed by experienced personnel to minimize distress. Analgesics were not routinely administered due to potential interference with EAE pathology; instead, strict humane endpoints were applied to prevent undue suffering.

Mice were euthanized upon reaching any of the following criteria: a loss of ≥20% of initial body weight, development of severe hind limb paraparesis resulting in impaired ability to access food or water, evidence of persistent self-inflicted injury at injection sites (e.g., repeated scratching or chewing leading to open lesions), lack of food or water intake for 24 h, or signs of severe respiratory distress (e.g., cyanosis, dyspnea). Upon reaching any of the defined humane endpoints, mice were immediately euthanized using ketamine/xylazine anesthesia followed by cervical dislocation. No animals died prior to reaching predefined humane endpoints or their scheduled experimental euthanasia.

All personnel involved in animal care and experimental procedures were certified according to FELASA category B standards. In addition, staff participated in regular continuing education and training to maintain and update their competencies in animal handling and welfare.

In total, 63 mice were used in the experiments. The method of euthanasia depended on the experimental requirements. For the acquisition of RNA or splenocytes, mice were anesthetized by intraperitoneal injection of ketamine (80 mg/kg body weight) and xylazine (8 mg/kg body weight), followed by cervical dislocation to ensure death. For histological analyses, mice were deeply anesthetized by intraperitoneal ketamine/xylazine and transcardially perfused with 4% paraformaldehyde.

### EAE induction and DRF treatment

10–12-week-old female C57BL/6J mice were subcutaneously injected with 200 μg myelin oligodendrocyte glycoprotein (MOG35–55; Genaxxon Bioscience GmbH, Germany) and 1 mg Complete Freund’s Adjuvant (BD/Difco, USA) containing 2 mg/ml Mycobacterium tuberculosis (H37RA; BD, USA) under ketamine/xylazine anesthesia. Mice received 200 ng Pertussis toxin (List Labs, USA) intraperitoneally on the day of immunization and two days later. Clinical symptoms were assessed daily in a blinded manner according to a 5-point scoring system, and animals were concurrently monitored regarding predefined humane endpoints. Depending on the experimental setup, we started daily oral gavage of DRF (90 mg/kg) dissolved in methocel or methocel (methylcellulose; negative control) in a volume of 200µl once per day when clinical symptoms of EAE manifested between d12 and d16 post immunization (p.i.; therapeutic experiment) or starting on the day of immunization (d0 p.i.; preventive experiment). For therapeutic experiments, mice were randomized based on EAE severity for allocation to either the DRF or methocel group.

Mice were maintained for a maximum of 50 days post immunization (d50 p.i.), at which point experiments were terminated and animals were euthanized.

For a matched pairs analysis, mice in the DRF and methocel groups were matched according to their EAE scores on the second day of clinically observable deficits. Differences in EAE severity over the course of the disease in the matched pairs were analyzed using the Wilcoxon matched-pairs signed rank test.

### Histology

Mice were transcardially perfused with 4% paraformaldehyde (PFA, Sigma) at day 50 (chronic stage of disease). Spinal cords were removed, postfixed for 3−4 h in 4% PFA and paraffin embedded. The tissue was sectioned with a microtome into 5 µm transversal slices. The slides were dewaxed and rehydrated, followed by antigen retrieval by boiling them in 1 mM EDTA (pH 8) for 38 min. After letting the tissue cool down, endogenous peroxidases were blocked with 0.5% H_2_O_2_ in 71% methanol with 0.2M NaN_3_. Afterwards, the slides were blocked with 10% BSA in PBS and incubated overnight with primary antibodies diluted in antibody diluent (DCS) as follows: rat CD3 1:200 (MCA147, clone CD3−12, Bio-Rad Laboratories), mouse 2,’3’-cyclic nucleotide 3’ phosphodiesterase (CNPase) 1:1000 (MAB326R, clone 11-5B, Merck Millipore), mouse glial fibrillary acidic protein (GFAP) 1:500 (644702, clone 2E1.E9, Biolegend), rat macrophage-3 antigen (MAC-3) 1:150 (550292, clone M3/84, BD), mouse pan neuronal marker 1:9000 (MAB2300, Merck), rabbit neurite outgrowth inhibitor A (NogoA) 1:1000 (AB5888, polyclonal, Merck Millipore), rabbit oligodendrocyte transcription factor 2 (Olig2) 1:500 (AB9610, polyclonal, Merck Millipore). After washing, the tissue sections were incubated with 1:200 biotinylated goat anti-mouse IgG (BA9200, Vector Laboratories), goat anti-rabbit IgG (BA1000, Vector laboratories) or rabbit anti rat IgG (BA4001, Vector laboratories) for 45 min at room temperature (RT). For immunodetection the slides were incubated with avidin-coupled peroxidase for 35 min at RT (Vectastain Elite Avidin-Biotin-Complex Kit, Vector laboratories). Di-amino-benzidine (DAB) was used as chromogen and hematoxylin was used as a counterstaining for nuclei visualization.

### Microscopy

Quantification of immune cell infiltration, myelination and glial/neuronal cells was performed by blinded observers in 9 independent sections of the spinal cord per EAE mouse, three segments each from cervical, thoracic and lumbar regions of spinal cord. Densities of T cells and macrophages were assessed in the biggest infiltrates per section, density was calculated based on counts in an area of 0.0625 mm^2^ by laying a stereological grid over sections. Counting of glial cells was performed equally standardized in the lateral and anterior column of spinal cord white matter. Proportion of demyelinated area is given in percent and was calculated by measuring areas of spinal cord white matter in ImageJ. [19] For assessment of neuronal cells, large alpha motoneurons were counted in the anterior horn. Relative axonal density was quantified by a 100µm diameter grid [20] in anterior and lateral column of spinal cord white matter.

Images were taken with a Leica DMR microscope using the Leica DFC320 camera and the Las X software. Saved images in the TIF format had a resolution of 1.59 pixels per μm and 3.24 megapixels.

### RNA isolation and quantitative real-time PCR

RNA from splenocytes was isolated using the RNeasy Kit (Qiagen, Germany), while RNA from brain tissue was extracted with TRIzol reagent (Thermo Fisher Scientific, USA), both according to the manufacturers’ instructions. RNA yield was quantified by absorbance measurements at 260 nm. Reverse transcription was performed by using the GoScript Reverse Transcription Mix Oligo(dT) (Promega). PCR reactions were performed on a qTOWER^3^-real-time-thermocycler (Analytik Jena, Jena, Germany) in triplicates. Relative quantification was performed by the ΔΔCT method, normalizing target gene expression to β-Actin as a housekeeping gene. The following TaqMan® real-time PCR assays (Thermo Fisher Scientific, USA) were used: Actb (Mm00607939_s1), Akr1b8 (Mm00484314_m1), Csf2 (Mm01290062_m1), Foxp3 (Mm00475162_m1), Hmox1 (Mm00516005_m1), Ifng (Mm01168134_m1), Il4 (Mm00445259_m1), Il10 (Mm01288386_m1), Il17a (Mm00439618_m1), Keap1 (Mm00497268_m1), Nqo1 (Mm01253561_m1), Nrf2 (Mm00477784_m1), Stat4 (Mm00448881_m1), Tbx21 (Mm00450960_m1), Tnf (Mm00443258_m1).

### Isolation of splenic cells

Spleens were removed on day 10 of EAE and disrupted with a 5 ml glass homogenizer. Cells were filtered through a 100 µM cell strainer followed by erythrocyte lysis. Cells were washed with cold DPBS and used for flow cytometry analysis or the MOG_35–55_ restimulation assay.

### Isolation of CNS infiltrating cells

Spinal cord tissue was removed on day 18 p.i. after perfusion with cold DPBS and disrupted with a 5 ml glass homogenizer. Cells were transferred to a Percoll^TM^ density gradient and centrifuged at 800g for 20 min without break. Cells at the interphases were collected, washed with cold DPBS and analyzed by flow cytometry.

### Flow cytometry

Splenic and CNS-infiltrating cells were obtained *ex vivo* and stained for extra- and intracellular markers. Identification of dead cells was achieved using a fixable viability dye, eFluor®780 (0.2 μl/test, eBioscience). Non-specific Fc mediated interactions were blocked by the addition of 0.5 μl anti-CD16/32 (93, eBioscience) for 10 min. For surface staining, cells were incubated with the below stated fluorochrome-conjugated antibodies for 30 min at 4°C. For intracellular cytokine staining, cells were stimulated for 4 h with ionomycin (1 μM) and phorbol-12-myristate-13-acetate (PMA, 50 ng/ml, both Sigma-Aldrich, USA) in the presence of monensin (2 μM, eBioscience), and made permeable with the Foxp3/Transcription Factor Staining Buffer Set (eBioscience/Thermo Fisher Scientific, USA). Intracellular cytokines were stained with the respective fluorochrome-conjugated antibodies for 45 min at 4°C. The following antibodies were used: CD3 (145-2c11, BD Biosciences), CD4 (RM4–5, eBioscience), CD25 (PC61, BioLegend), FoxP3 (FJK-16s, eBiosciences), IL-17A (eBio17B7, eBioscience), IL-10 (JESS-16E3, BioLegend), IFN-γ (XMG1.2, BD Biosciences). Probes were measured with a flow cytometer (FACS CantoII, BD Biosciences), data were analyzed with FlowJo software (BD Biosciences).

### MOG peptide recall assays and cytokine measurement

Splenocytes were harvested on day 10 p.i. from DRF and methocel treated EAE mice. Splenocytes were stimulated for 3 days with MOG_35−55_ peptide (20 µg/ml or 100 µg/ml) or Concanavalin A (1,25 µg/ml). Afterwards, supernatants were collected and levels of IFNγ, IL-17A, IL-10, IL-4 and GM-CSF were measured using enzyme-linked immunosorbent assays (ELISA) kits (DuoSet ELISA Kits, R&D Systems, Minneapolis, MN) as per the manufacturer’s guidelines.

### Statistical methods

All statistical tests were performed using GraphPad Prism (GraphPad Software, USA). For the analysis of clinical severity in EAE, we used the Mann–Whitney U test to compare disease scores on single days between groups. To compare the overall disease course, a mixed-effects model (restricted maximum likelihood, Geisser–Greenhouse correction) was applied. For the matched-pairs analysis of clinical course, the Wilcoxon matched-pairs signed-rank test was performed. For histological data and cytokine concentrations in supernatants, the Mann–Whitney U test was employed. Data from flow cytometry and quantitative real-time PCR at different time points during MOG-EAE were analyzed using a 2-way ANOVA with Fisher’s LSD. Given the exploratory nature of the flow cytometry and qPCR analyses, no correction for multiple comparisons was applied.

## Results

### DRF treatment ameliorated the clinical course of MOG-EAE

**Fig 1:**
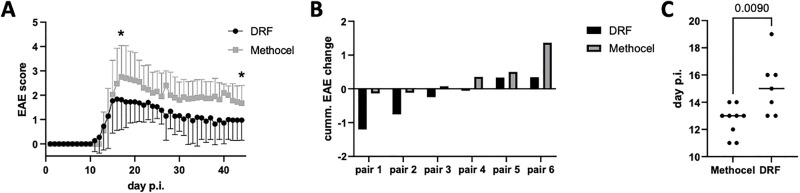
DRF treatment ameliorated the course of EAE in acute and chronic stages. MOG_35–55_-EAE was induced in 12 week old C57BL/6J mice. (**A**) Oral gavage of vehicle (methocel) or DRF (90 mg/kg body weight) once daily was started on the day clinical signs of EAE developed, ranging between d12 and d16 p.i.. EAE score was obtained on a daily basis. Mice treated with DRF developed less severe EAE in acute and chronic stages of the disease (Mann-Whitney-U test d17 p = 0.0418, d44 p = 0.044, n(DRF)=12, n(Methocel)=10). In a mixed-effects model significant effects of treatment (p = 0.0408), and a significant time × treatment interaction (p = 0.0278) indicated that disease progression differed between treatment groups over time. (**B**) 12 mice that were therapeutically treated with DRF or methocel were matched based on their EAE score on the second day of clinically observable deficits. In the matched pairs analysis, DRF treated animals showed reduced EAE severity in the course of disease compared to vehicle controls. (Wilcoxon matched-pairs signed rank test p = 0,0313) (**C**) Preventive treatment with DRF resulted in a delayed onset of clinically observable deficits (paired t-test p = 0.009; n(DRF)=7, n(vehicle)=9).

To analyze the effect of DRF on MOG-EAE, we immunized C57BL/6J mice and started daily oral gavage of DRF or methocel (control) when clinical signs of EAE manifested (therapeutic approach) or started at day of immunization (preventive approach, day 0 p.i.). EAE diseased mice treated with DRF showed significantly lower EAE scores compared to the methocel treated group in the therapeutic approach (Fig 1A). Pairwise comparison of the disease course of mice with equal EAE scores on the second day of clinically observable deficits demonstrated that DRF improved the severity of disease (pairs 1–4) or prevented mice from developing severe EAE symptoms (pairs 5 and 6) (Fig 1B). In preventively DRF treated mice, onset of symptomatic EAE was delayed (Fig 1C), while reduced EAE severity was not significant.

### Therapeutic DRF treatment reduced immune cell infiltration and prevented myelin and axon loss during MOG-EAE

**Fig 2:**
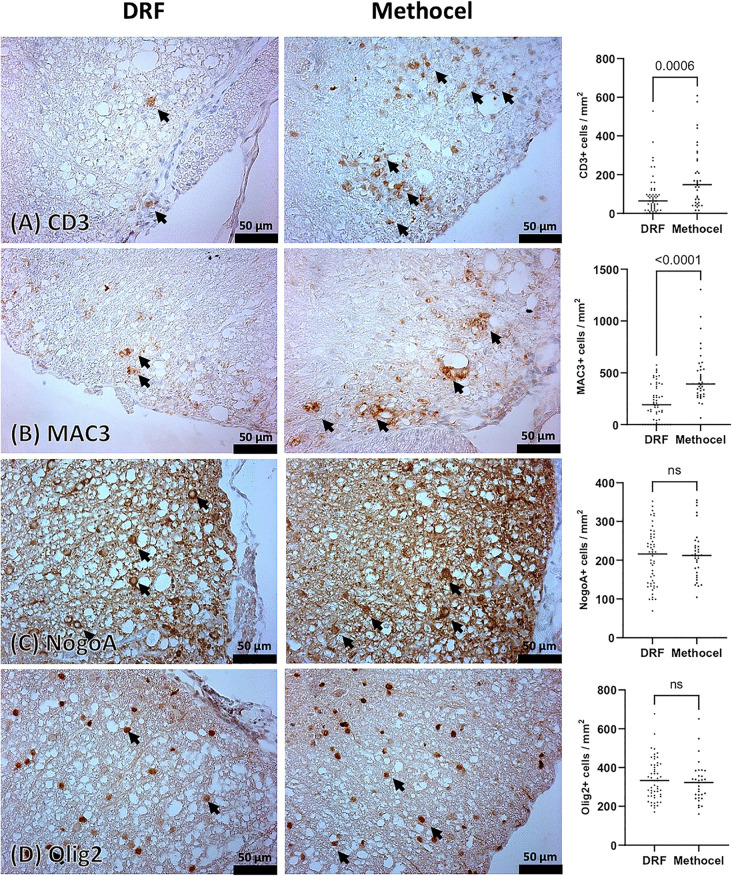
Histological correlation of DRF treatment in chronic EAE 1. Daily oral gavage of methocel or DRF (90 mg/kg) was started when clinical signs of MOG_35–55_-EAE appeared. Spinal cords were harvested on day 50 p.i. and histologically stained for (**A**) T cells, (**B**) macrophages, (**C**) mature oligodendrocytes and (**D**) oligodendroglial cells. Left: Representative images, right: quantification of the respective cell type presented as mean ± SEM. We used Mann-Whitney-U for statistical evaluation.

**Fig 3:**
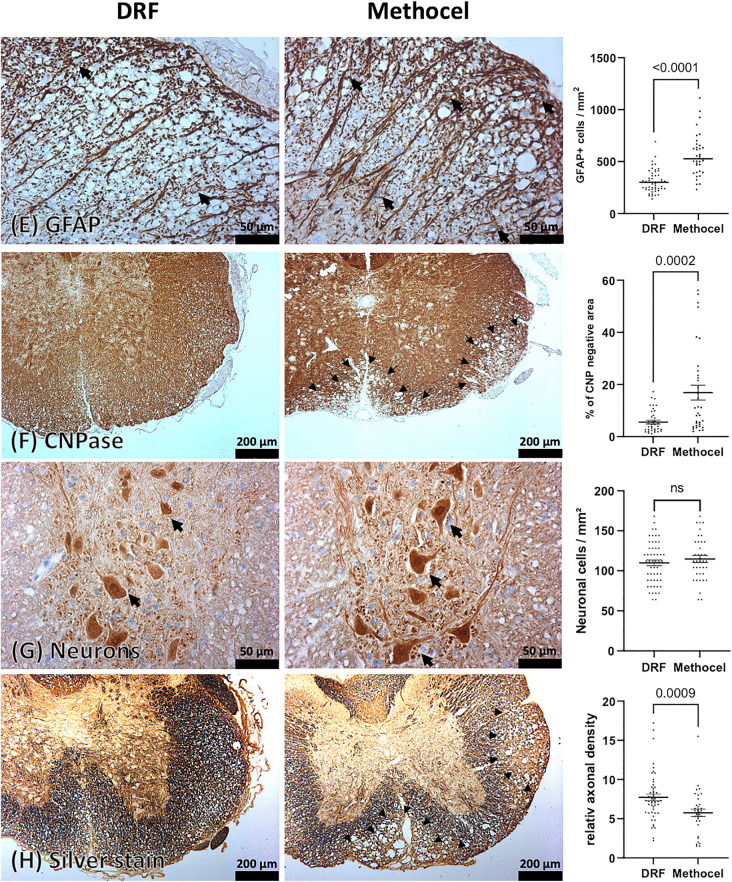
Histological correlation of DRF treatment in chronic EAE 2. Daily oral gavage of methocel or DRF (90 mg/kg) was started when clinical signs of MOG_35–55_-EAE appeared. Spinal cords were harvested on day 50 p.i. and histologically stained for (**E**) astrocytes, (**F**) myelin, (**G**) neurons and (**H**) axons. Left: Representative images, right: quantification of the respective cell type presented as mean ± SEM. We used Mann-Whitney-U for statistical evaluation.

To investigate on the impact of therapeutic DRF treatment on immune cell infiltration into the CNS as well as neuronal and axonal densities, myelination and changes in glial cells, spinal cords of EAE diseased mice were removed on day 50 p.i. and histologically analyzed. In spinal cords of DRF treated mice, we observed a significantly reduced number of infiltrating CD3 + T cells, which was accompanied by a lesser density of macrophages (Table 1, Fig 2A, 2B). Additionally, astrogliosis, as a pathologic hallmark of inflammatory CNS tissue damage, was reduced by 45% compared to control treated animals (Table 1, Fig 3E). In contrast, myelination in spinal cord white matter was maintained (Table 1, Fig 3F), and DRF-treated animals showed a higher axonal density compared to control animals (Table 1, Fig 3H). However, we did not observe differences in oligodendroglial and neuronal densities in DRF and methocel treated animals. (Table 1, Fig 2C, 3D and 3G)

**Table 1:** Histological correlation of therapeutic DRF treatment in chronic EAE.

	DRF	Methocel	P value (Mann-Whitney-U)
T cells (CD3 ^+^ cells/mm^2^ ± SEM)	89.63 ± 13.69	185.1 ± 26.13	0.0006
Macrophages/microglia (Mac-3 ^+^ cells/mm^2^ ± SEM)	217.3 ± 23.97	461.3 ± 41.71	< 0.0001
Mature oligodendrocytes (NogoA^+^ cells/mm^2^ ± SEM)	206.9 ± 9.96	215.5 ± 10.94	ns
Oligodendrioglial cells (Olig2 ^+^ cells/mm^2^ ± SEM)	337.2 ± 14.7	322.8 ± 18.97	ns
Demyelinated area (% of CNP^-^ white matter ± SEM)	5.59 ± 0.69	16.86 ± 2.84	0.0002
Astrogliosis (GFAP^+^ cells/mm^2^ ± SEM)	307.9 ± 14.84	559.6 ± 33.2	< 0.0001
Neuronal cells (cells/mm^2^ ± SEM)	109.9 ± 3.65	114.8 ± 4.45	ns
Relative axonal density ± SEM	7.72 ± 0.42	5.76 ± 0.46	0.0009

Daily oral gavage of methocel or DRF (90 mg/kg) was started when clinical signs of MOG_35-55_-EAE appeared. Spinal cords were harvested on day 50 of EAE and histologically analyzed for T cells, macrophages, mature oligodendrocytes, oligodendroglial cells, myelin, astrocytes, neurons and axons. Data are given as mean ± SEM and were analyzed by Mann-Whitney-U for statistical evaluation.

### Characterization of peripheral immune cells and spinal cord infiltrating cells in DRF treated mice

**Fig 4:**
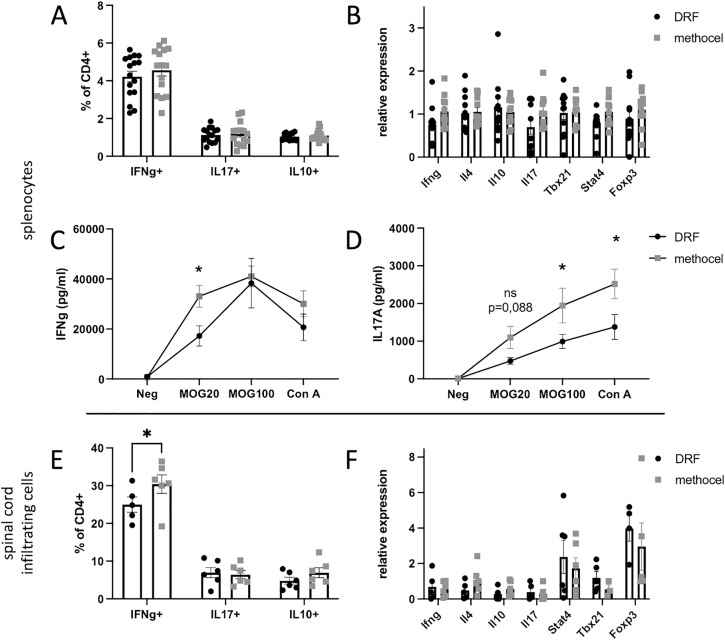
DRF treatment reduces Th1 responses during MOG-EAE (A) Splenocytes were isolated from EAE diseased mice at day 10 p.i. and analyzed by flow cytometry. No differences were measured between DRF and methocel treated animals (n = 16 vs 15). (B) RNA was isolated from splenocytes and analyzed *via* RT-qPCR for representative immune cell genes. There was no significant alteration of relative expression of stated genes at the inductive phase of EAE (n = 11 vs 11). (C, D) *In vitro* restimulation of isolated splenocytes with MOG_35–55_ peptide or ConA revealed (**C**) a reduced secretion of IFNγ (* p = 0.013) and (D) IL17 (* p = 0.028 for MOG100 and *p = 0.0158 for ConA) in splenocytes from DRF treated mice. (E) Spinal cord infiltrating cells were isolated by gradient centrifugation from EAE mice at day 18 p.i. treated with methocel or DRF (90 mg/kg body weight) once daily starting at day 0 p.i.. Flow cytometry analysis of different immune cell subsets revealed a reduced frequency of IFNγ^+^ T cells in spinal cords of DRF treated EAE mice. (F) RNA was isolated from spinal cord tissue at acute stages of EAE and analyzed *via* RT-qPCR for representative immune cell genes. There was no significant alteration of relative expression of stated genes at the maximum of EAE (n = 6 vs 6). (A,B,E,F) Data are presented as mean + SEM and were analyzed by 2-way ANOVA Fisher LSD. (C, D) Data are presented as mean + SEM and were analyzed by Mann-Whitney test.

To assess immunological alterations after DRF treatment, we performed flow cytometric analyses of splenocytes and spinal cord–infiltrating cells at different time points during MOG-EAE. We examined the frequencies of CD4⁺ and CD8 ⁺ T cells, FoxP3-expressing Tregs, IFNγ ⁺ Th1 cells, and IL-17 ⁺ Th17 cells. No differences in the frequencies of these cell populations were observed in splenocytes at the inductive stage of MOG-EAE (day 10 p.i.) between DRF- and methocel-treated mice (Fig 4A). To assess potential transcriptional effects of DRF, we further analyzed the mRNA expression of several immunologically relevant genes (*Ifng, Il4, Il10, Il17a, Foxp3, Stat4, Tbx21*) by quantitative real-time PCR. No significant differences in gene expression were detected in splenocytes upon DRF treatment (Fig 4B). However, stimulation of isolated splenocytes with MOG_35–55_ peptide resulted in reduced secretion of IFNγ and IL-17A in DRF-treated mice (Fig 4C,4D), while secretion of IL-4, IL-10, and GM-CSF was unchanged (supplementary figure S1 Fig).

For analysis of CNS-infiltrating immune cells at the peak of EAE, mice were treated with DRF or methocel starting on day 0 of immunization. On day 18 p.i., spinal cords were isolated and infiltrating cells were harvested by gradient centrifugation. We observed reduced frequencies of IFNγ⁺ cells in spinal cords of DRF-treated mice (Fig 4E). mRNA analysis of spinal cord tissue at acute (Fig 4F) and chronic stages (supplementary figure S2 Fig) of EAE by quantitative real-time PCR revealed no differences in the relative expression of the investigated genes between DRF- and methocel-treated mice.

### DRF treatment leads to an upregulation of Nrf2-associated genes in the spinal cord of EAE diseased mice

**Fig 5:**
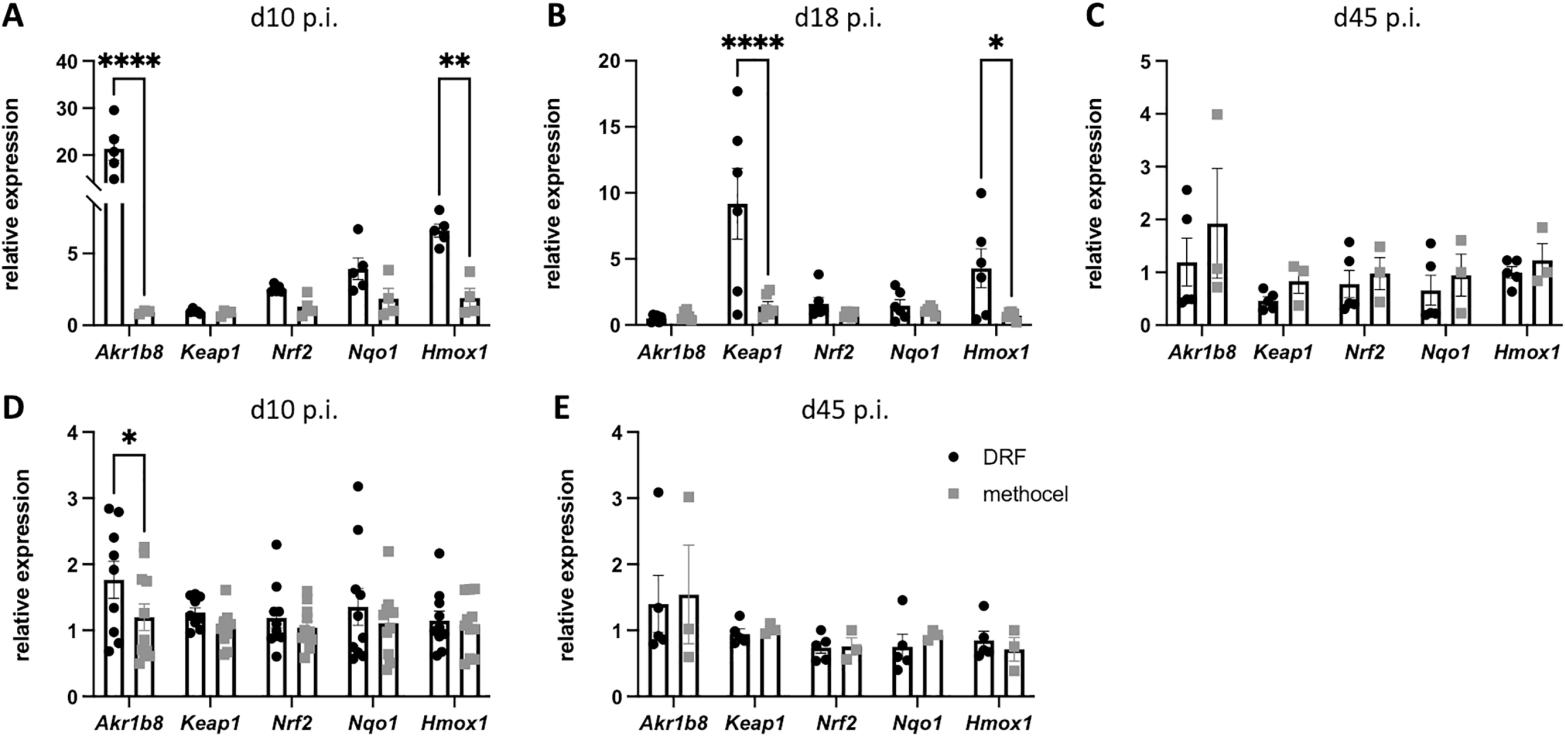
RNA analysis in spinal cord tissue and splenocytes during inductive, acute and chronic stages of EAE. Spinal cords were harvested from EAE diseased mice on day (**A**) 10, (**B**) 18 and (**C**) 45 p.i. which received vehicle or DRF (90 mg/kg body) starting at day 0 p.i. Splenocytes were harvested on day (**D**) 10 and (**E**) 45. Nrf2 target genes were upregulated early after beginning of oral gavage of DRF, especially in the spinal cord, whereas in chronic stages, there was no increased expression of Nrf2 target genes in DRF treated mice compared to control mice. Data are presented as mean + SEM and were analyzed by 2-way ANOVA Fisher LSD for statistical evaluation. (2-way ANOVA, with Fishers LSD test; for A, D n = 11 vs 11; for B n = 6 vs 6; for C, E n = 3(Methocel) vs 5(DRF)).

Fumaric acid esters are known for leading to disinhibition of Nrf2 and increased expression of Nrf2 target genes [7] To assess the effects of DRF treatment during MOG-EAE on Nrf2 target genes, we isolated RNA from spinal cords and splenocytes at different time points of the disease. Quantitative real-time PCR revealed increased expression of *Akr1b8* and *Hmox1* in the spinal cords of DRF-treated mice during the inductive stages of EAE, with a borderline-significant trend towards increased expression of *Nqo1* and *Nrf2* (Fig 5A). In the acute stages of MOG-EAE, we observed upregulation of *Keap1* and sustained increased expression of *Hmox1* after DRF treatment, whereas no differences were observed during chronic EAE (Fig 5B, 5C). Differences of expression in Nrf2 target genes in splenocytes during inductive stages of EAE was only significant for *Akr1b8* expression, and the difference was not as pronounced as in spinal cords of the same animals (Fig 5D). In splenocytes harvested at a chronic stage of EAE, we did not detect differences in DRF and methocel treated mice (Fig 5E), suggesting that Nrf2 signaling is predominantly involved during early EAE.

## Discussion

Our data demonstrate that orally administered DRF exerts therapeutic effects in the MOG-induced EAE model, associated with reduced Th1 responses, diminished CNS infiltration, and robust upregulation of Nrf2 target genes in the CNS.

DRF treatment resulted in an ameliorated disease course with less severe maximum of disease symptoms, indicating a potent therapeutic effect of DRF during experimental neuroinflammation (Fig 1A). Compared to the study by Yadav et al., in which DRF was administered from day 0 of EAE induction, we demonstrate therapeutic effects even when treatment is initiated at the onset of clinical signs [21]. However, the reduction in EAE scores was less pronounced in our cohort.

DRF treated animals were less affected from EAE symptoms also in chronic stages of the disease, as it has already been reported for DMF [7,10]. Moreover, we could show a delayed onset of the disease when DRF was administered preventively, suggesting an early modulatory effect of the compound during neuroinflammation (Fig 1C).

Indeed, we observed a reduced Th1 immune response in the priming phase of EAE before the onset of first clinical signs, supporting an immunomodulatory role of DRF. For analysis of alterations in the immune response to MOG immunization, we performed MOG recall assays in preclinical stages of EAE. DRF treatment led to a reduced secretion of IFNγ and IL-17 from splenocytes (Fig 4C, 4D). Our findings are in line with previous research for fumaric acid esters in murine models [10,21] and in people with multiple sclerosis [9,22,23], which reports a skew of the immune response towards a Th2 phenotype with suppression of Th1 and Th17 responses. At later stages of the disease, histology demonstrated a reduced spinal cord infiltration of T cells (Fig 2A), as it was described for other fumaric acid esters in MOG-EAE [24], but also in a focal EAE-model [25]. Further characterization of spinal cord infiltrating cells revealed decreased frequencies of CD4^+^IFNγ^+^ T cells at the maximum of EAE (Fig 4E), thus supporting our observation of a less pronounced Th1 cell response after DRF treatment. Moreover, the beneficial effect of DRF administration also involved a reduced infiltration of macrophages to the CNS, coinciding with reduced astrogliosis and demyelination, while axonal densities were preserved in DRF treated mice (Figs 2 and 3). Similar findings were reported for DMF, however, we did not detect a reduced loss of neuronal cells in the spinal cord, as was reported for the treatment of EAE diseased mice with DMF [7,24].

The reduced infiltration of immune cells to the CNS may be due to a decreased expression of CNS-homing receptors, as it has been described for DRF by Yadav et al [21] and observed in CD4 T cells of DMF treated MS patients [22]. Yet, we did not investigate a potential effect of DRF on T cell migration within this study. DMF has been shown to contribute to the integrity of the blood-brain barrier in the context of CNS hypoxia [26] Since blood-brain barrier integrity is also crucial in neuroinflammation, this could represent another mechanistic link, resulting in reduced immune cell infiltration after treatment with fumaric acid esters.

Besides a direct immunomodulatory effect, DRF may also possess neuroprotective mechanisms by modulating cation channels and reducing hyperexcitability, as was shown in a cuprizone mouse model of toxic demyelination [27].

The best-characterized neuroprotective effects of fumaric acid esters are mediated by activation of the Nrf2 pathway within the CNS [6,7].

In our experiments, upregulation of the Nrf2 pathway was pronounced in CNS tissue (Fig 5A-5C): we measured a strong upregulation of the Nrf2 target genes *Akr1b8* and *Hmox1* 10 days after the start of DRF treatment. In the following, *Keap1* was upregulated in mice treated with DRF, while in chronic stages of EAE, i.e., after 45 days of DRF treatment, no differences in Nrf2 associated genes were detectable in the CNS. These dynamic changes may reflect overlapping effects, as Nrf2 bidirectionally interacts with other transcription factors (HIF1a, NOTCH1, aryl hydrocarbon receptor), which share partially overlapping target gene profiles [28–30]. The observed upregulation of *Keap1* may be interpreted in the context of an autoregulatory feedback loop, as described by Lee et al. [31]. The absence of differences in chronic stages of the disease may reflect a transcriptional steady state at this time point.

On a protein level, an increased expression of Nrf2 in neuronal and glial cells upon long term DMF treatment [7], as well as antioxidative and neuroprotective effects were described *ex vivo* [8] and *in vivo* [7,32]. The relevance of DRF induced Nrf2 signaling in oligodendroglial cells was demonstrated in the context of ferroptosis, where DRF treatment limited ferroptotic oligodendrocyte and myelin damage [33]. Interestingly, DRF was reported to reduce mechanical hypersensitivity in wild-type but not Nrf2 knockout mice in a model of neuropathic pain, suggesting a potential role for Nrf2-dependent mechanisms in the peripheral nervous system [34].

In contrast, in PBMCs we only detected differences in *Akr1b8* expression at an early timepoint (d10), with no significant differences to control animals in the chronic stage of the disease (Fig 5D, 5E). This is comparable to an analysis of differentially expressed genes in MS patients receiving DMF: Here PBMCs initially showed an upregulation of Nrf2 associated genes, while gene expression normalized as early as 6 weeks after the beginning of the therapy [35]. Irrespective of these findings, relevance of Nrf2 pathway upregulation by fumaric acid esters in T cells remains unclear, as inhibition of Nrf2 signaling neither altered metabolic effects, nor induction of apoptosis in T cells after DMF treatment [36] and immunomodulation with suppression of Th1 and Th17 responses was also described in Nrf2 knockout mice [10]. While NFkB was shown to be inhibited by Nrf2 activation [37], fumaric acid esters are also described to directly block NFkB signaling via covalent modification of p65, independently of Nrf2 [38].

Beyond its effects on mature immune cells, Nrf2 also influences hematopoietic stem cell homeostasis, migration and bone marrow retention, partly through modulation of CXCR4 signaling [39]. Whether activation of this pathway by fumaric acid esters contributes to their immunomodulatory effects remains to be determined.

In summary, oral administration of DRF in the MOG-induced EAE model resulted in immunomodulation characterized by reduced Th1 and likely Th17 responses, decreased CNS immune cell infiltration and demyelination, and preservation of axonal integrity, ultimately leading to improved clinical outcomes. Mechanistic analyses suggest involvement of Nrf2-associated pathways within the CNS; however, the immunomodulatory effects of DRF may not be fully dependent on Nrf2 activation, indicating both overlapping and distinct mechanisms of action.

Further studies are needed to disentangle the relative contributions of Nrf2-dependent and Nrf2-independent pathways to the immunomodulatory and neuroprotective effects of DRF.

### Disclosures

DF, AS, AK have nothing to disclose, RG has received research support or honoraria from Bayer Schering, Biogen-Idec, BMS, Chugai, Eisai, ELAN, Janssen, Kyverna, Merck Serono, Neuraxpharm, Nikkiso Pharma, Novartis, Roche, Sanofi-Genzyme, TEVA, ZLB Behring, Baxter, Talecris, RL received personal compensations from Merck, Sanofi and Novartis. SH received research support from Novartis. This does not alter our adherence to PLOS ONE policies on sharing data and materials.

## Supporting information

S1 Fig Splenocytes harvested at day 10 p.i. from DRF treated mice showed no differences in secretion of IL-4, IL-10 or GM-CSF after in vitro restimulation with MOG_35–55_ peptide or ConA compared to methocel treated mice. (TIF)

S2 Fig RNA was isolated from spinal cord tissue at chronic stages of EAE and analyzed *via* RT-qPCR for representative immune cell genes. (TIF)

S1 Appendix Raw data on clinical course of EAE, histology, cytokine analysis, flow cytometry, qPCR. (XLSX)
